# Systemic Mastocytosis with Smoldering Multiple Myeloma: Report of a Case

**DOI:** 10.1155/2016/3161768

**Published:** 2016-05-16

**Authors:** Sassine Ghanem, Gwenalyn Garcia, Liu Ying, Matthew Hurford, Marcel Odaimi

**Affiliations:** ^1^Department of Medicine, Staten Island University Hospital, Northwell Health, 475 Seaview Avenue, Staten Island, NY 10305, USA; ^2^Department of Hematology/Oncology, Staten Island University Hospital, Northwell Health, 475 Seaview Avenue, Staten Island, NY 10305, USA; ^3^Department of Pathology, Staten Island University Hospital, Northwell Health, 475 Seaview Avenue, Staten Island, NY 10305, USA

## Abstract

Systemic mastocytosis (SM) is a disease characterized by a clonal infiltration of mast cells affecting various tissues of the body. It is grouped into six different subtypes according to the World Health Organization classification. It is called indolent systemic mastocytosis (ISM) when there is no evidence of end organ dysfunction, while the presence of end organ dysfunction defines aggressive systemic mastocytosis (ASM). When SM coexists with a clonal hematological disorder, it is classified as systemic mastocytosis with associated clonal hematological nonmast cell lineage disease (SM-AHNMD). Over 80% of SM-AHNMD cases involve disorders of the myeloid cell lines. To our knowledge, there are only 8 reported cases to date of SM associated with a plasma cell disorder. We report a patient with ISM who was found to have concomitant smoldering multiple myeloma. His disease later progressed to ASM. We discuss this rare association between SM and a plasma cell disorder, and potential common pathophysiologic mechanisms linking the two disorders will be reviewed. We also discuss prognostic factors in SM as well as the management options considered during the evolution of the patient's disease.

## 1. Introduction

Mastocytosis is a disease characterized by the abnormal infiltration of clonally derived mast cells in different tissues of the body. When the infiltration is limited to the skin, a diagnosis of cutaneous mastocytosis is made. Extracutaneous infiltration of clonal mast cells defines systemic mastocytosis (SM). It typically involves the bone marrow but can also affect any other organ [1].

The classification for systemic mastocytosis was established by Valent et al. [1] in a consensus proposal and later adopted in the World Health Organization 2008 classification [2]. It recognizes six different groups of SM: indolent systemic mastocytosis (ISM), systemic mastocytosis with associated clonal hematological nonmast cell lineage disease (SM-AHNMD), aggressive systemic mastocytosis (ASM), mast cell leukemia, mast cell sarcoma, and extracutaneous mastocytoma. The difference between indolent and aggressive SM depends on the presence of C findings, which indicate organ dysfunction secondary to excessive mast cell infiltration. C findings include gastrointestinal malabsorption, hypersplenism, hepatic dysfunction, cytopenias, and pathologic fractures [1, 2].

In a retrospective study of 66 patients with SM, Travis et al. found SM-AHNMD to be the second most common subtype (after ISM), with 22 patients affected. Over 80% of SM-AHNMD cases involved disorders of the myeloid cell lines [3].

To our knowledge, there are only 8 reported cases to date of SM associated with a plasma cell disorder (Table 1) [4–11]. We present a patient with ISM with concomitant smoldering multiple myeloma whose disease later progressed to ASM.

## 2. Case Presentation

A 59-year-old male with a known history of ISM incidentally found on pathologic review after surgery for osteoarthritis was referred to the hematology clinic for evaluation of suspected monoclonal gammopathy. His past medical history was significant for splenectomy. The exact indication for the procedure was unclear, but it was possibly done for involvement with lymphoma versus mastocytosis. He presented with suspected monoclonal gammopathy on previously done blood tests with the results shown in Table 2. His physical exam revealed no abnormalities.

Repeat serum protein electrophoresis and immunofixation again revealed an IgG kappa monoclonal protein. The M-spike was 3.8 g/dL. The serum IgG level was elevated at 4430 mg/dL, while IgA and IgM levels were within the normal range. Serum free kappa light chains were elevated at 203.1 mg/L with normal free lambda light chains of 9.4 mg/L and a free kappa/lambda ratio of 21.61. LDH was normal at 145 IU/L and beta-2 microglobulin was elevated at 3.8 mg/L. The tryptase level was elevated at 76 ng/mL.

**Figure 1:**
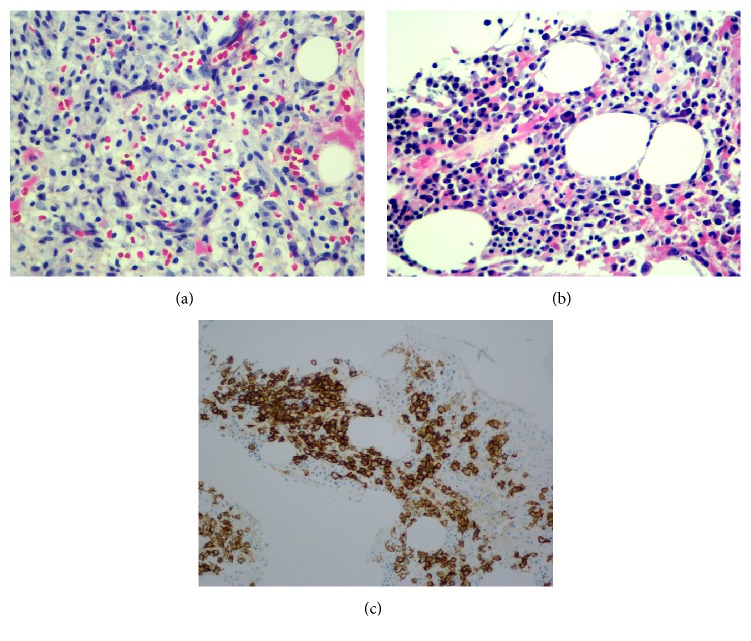
Bone marrow biopsy showing an infiltrate of spindle-shaped mast cells (a) comprising 50% of the marrow. Plasma cell infiltrate (b) occupying 30% of the remaining marrow, with an image showing CD138 staining (c).

Bone marrow aspiration and biopsy was performed, which showed an overall cellularity of 60–90%. Approximately 50% of the marrow was comprised of nodules of small mononuclear spindle cells (Figure 1(a)). Immunostains were positive for CD68, CD45, CD117, CD30, CD25, and M-tryptase but negative for CD56, consistent with a mast cell phenotype.

Plasma cells occupied at least 30% of the remaining marrow (Figure 1(b)). They were CD138 positive by immunohistochemistry (Figure 1(c)) and kappa restricted by* in situ* hybridization. Flow cytometry of the bone marrow aspirate detected a monoclonal IgG kappa plasma cell population comprising 4.7% of total cells in the sample. Cytogenetics testing revealed a normal male karyotype negative for mutations associated with multiple myeloma. Amyloid staining was negative.

Based on the above-mentioned morphology and immunohistochemical and molecular studies, in addition to the fact that the patient had no anemia, a normal calcium level, and no bone pain, a final diagnosis of ISM associated with smoldering multiple myeloma was established. The patient was followed up without specific treatment for either condition. An abdominal fat pad biopsy was done to rule out secondary amyloidosis as the cause of his chronic kidney disease, which came back negative.

Two years later, the patient presented to the emergency department with a 2-day history of generalized weakness, shortness of breath, and melena. A nasogastric lavage was done and showed bloody fluid. CBC on admission showed hemoglobin of 5.9 g/dL; WBC and platelet counts were within normal limits. The patient's coagulation parameters revealed a prolonged PT of >40 sec with an INR of >15 and a prolonged PTT of 65.6 sec. Of note, the patient was on anticoagulation with warfarin for atrial fibrillation. His transaminases and total bilirubin levels were within normal limits.

The patient received packed red cell and fresh frozen plasma transfusions. Emergent upper endoscopy showed grade IV gastroesophageal varices requiring band ligation. Severe hypertensive portal gastropathy was also found.

The patient had no known history of or obvious risk factors for liver cirrhosis; hence, workup was initiated for possible causes of portal hypertension. Ultrasound of the abdomen showed a normal sized liver with smooth contour and normal echogenicity. The portal and hepatic veins were both patent. There was small volume abdominal ascites. Screening for viral hepatitis was negative. Serum ceruloplasmin, alpha-1 antitrypsin, and ferritin levels were within normal limits. Testing for c-ANCA, p-ANCA, antimitochondrial, and antismooth muscle antibodies was negative.

**Figure 2:**
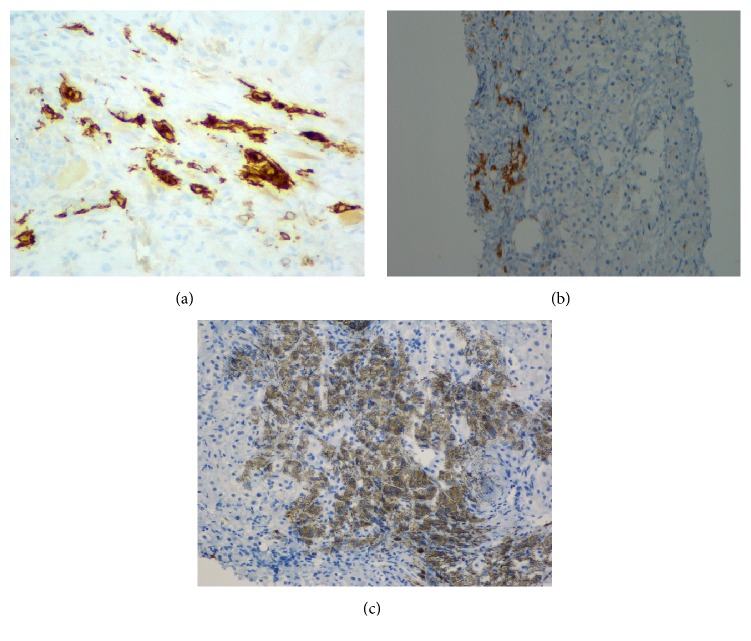
Liver biopsy immunohistochemical staining showing mast cells positive for CD117 (a), CD25 (b), and mast cell tryptase (c). Of note, (c) is zoomed in on the portion of the liver that is positive for mast cells.

Subsequently, liver biopsy was performed, which showed portal and periportal fibrosis with mild inflammation, compression of the portal veins, broad fibrous septa, and severe perisinusoidal fibrosis, confirmed by trichrome and reticulin stains. There was focal nodule formation. CD117 immunostaining demonstrated abundant mast cells in portal tracts. Rare cells were positive for CD25 and mast cell tryptase (Figure 2). Serum tryptase level was 104 ng/mL. These findings were consistent with mastocytosis involving the liver, resulting in extensive fibrosis. The patient's disease had progressed to ASM, and a course of treatment with cladribine as outpatient was recommended.

**Figure 3:**
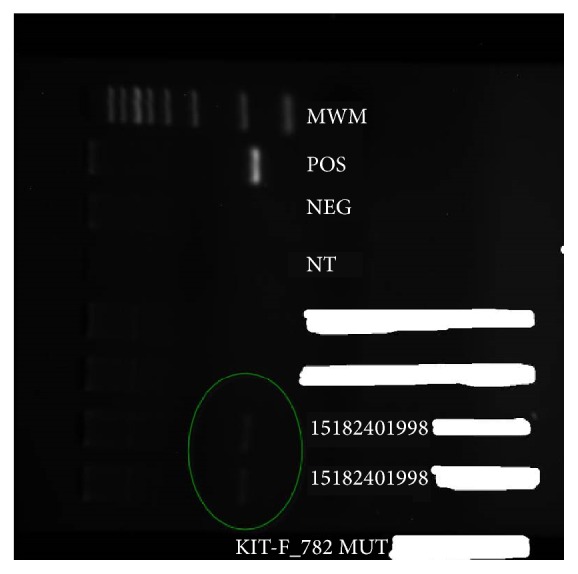
Sequence analysis of the KIT D816V mutation.

Subsequently, the patient followed up at a different institution where further evaluation showed a negative JAK 2 mutation and a positive KIT D816V mutation (Figure 3). A kidney biopsy was also performed to rule out secondary amyloidosis or multiple myeloma as the etiology of his chronic kidney disease; this showed interstitial fibrosis and tubular atrophy as well as features of thrombotic microangiopathy without any amyloid or immune-type deposits. The treating team however linked the chronic kidney disease to multiple myeloma and therefore started him on a treatment regimen of cyclophosphamide, bortezomib, and dexamethasone as well as hydroxyurea for the systemic mastocytosis. The patient tolerated therapy and only has complaints of fatigue in the days that follow the chemotherapy session.

## 3. Discussion

Criteria for the diagnosis of SM include 1 major criterion and 4 minor criteria. The major criterion is a multifocal dense infiltrate of mast cells (≥15 mast cells in aggregates) in sections from bone marrow and/or extracutaneous organs. The 4 minor criteria are as follows: (1) >25% of the mast cells in the infiltrate with spindle-shaped or atypical morphology or >25% of all mast cells in bone marrow aspirate smears characterized as immature or atypical, (2) presence of an activating point mutation at codon 816 of KIT, (3) expression of CD2 and/or CD25 in mast cells, and (4) total serum tryptase levels persistently exceeding 20 ng/mL. The first 3 minor criteria apply to samples obtained from blood, bone marrow, or other extracutaneous organs. The last criterion is not valid if there is an associated clonal myeloid disorder. The presence of the major criterion and 1 minor criterion or the presence of 3 minor criteria is required to establish the diagnosis of SM [2].

On evaluation in our clinic, the patient had a tryptase level of 76 ng/mL and a bone marrow biopsy showing CD25+ spindle-shaped mast cells occupying approximately 50% of the bone marrow, fulfilling the diagnostic criteria for SM. His bone marrow biopsy also showed CD138+ plasma cells occupying 30% of the remaining bone marrow. With this bone marrow finding along with a serum M-spike of 3.8 g/dL and the absence of end organ damage, the patient was diagnosed with concomitant smoldering multiple myeloma.

The coexistence of SM with multiple myeloma places our patient in the SM-AHNMD subtype. While associated myeloid/myelomonocytic neoplasia in SM-AHNMD accounts for 82%–89% of SM-AHNMD, associated lymphoproliferative disorders are rare [3, 4]. In their study of 138 cases of SM-AHNMD, Pardanani et al. found 7 patients (5.1%) with lymphoma, 5 patients (3.6%) with myeloma, and 2 patients (1.5%) with chronic lymphocytic leukemia (CLL) [12].

Since multiple myeloma occurring in an elderly patient is not uncommon, one could argue that the cooccurrence of SM with smoldering multiple myeloma was a coincidence. However,* in vitro* studies have shown the capacity of neoplastic mast cells to induce the growth of lymphocytic neoplasms. Tournilhac et al. demonstrated that the human mast cell line HMC-1 stimulated proliferation of the malignant lymphoplasmacytic cells of patients with Waldenstrom's macroglobulinemia through interactions between CD154 on the mast cells and CD40 on the lymphoplasmacytic cells [13].* In vitro* studies also suggest a role of mast cells in the growth of Hodgkin lymphoma via their expression of CD30 ligand [14]. A similar relationship may exist between mast cells and plasma cells as well. Mast cells secrete multiple cytokines including IL-6 and stem cell factor, both of which have been shown to induce plasma cell proliferation [15–17].

Whether or not the neoplastic mast cells of SM and the associated hematologic malignancy are derived from the same clone has been examined. In 2 cases of SM associated with lymphoproliferative disorders, the D816V mutation was detected in the neoplastic mast cells but not in the malignant lymphocytes, suggesting that the SM and the coexisting lymphoid malignancy are clonally distinct [18, 19]. In contrast, in cases of acute myeloid leukemia, the leukemic translocation t(8;21) can be detected in the malignant myeloid cells as well as in the neoplastic mast cells. Therefore, they are presumed to have developed from the same clone [20, 21].

When confronted with cases of SM-AHNMD, the current strategy is to treat each entity on its own as if the other entity did not coexist in the patient [22–24]. With an M-spike greater than 3 g/dL but no myeloma-defining event such as anemia, hypercalcemia, bony disease, or renal insufficiency secondary to myeloma, our patient fit into the diagnosis of smoldering multiple myeloma, and management of his disease was close clinical monitoring for transformation to symptomatic multiple myeloma.

In SM, the difference between indolent and aggressive disease depends on the presence of C findings, which indicate organ dysfunction related to mast cell invasion. The patient's left hip replacement was performed for degenerative joint disease and no pathological fracture had occurred. Therefore, the finding of mast cells in the surgical specimen was incidental. In addition, the pathological finding of mast cells on the bone marrow biopsy was also an incidental finding during workup of the patient's hypergammaglobulinemia. Symptoms associated with mast cell degranulation, such as urticaria, flushing, or diarrhea, were also not present in the patient's history. The patient was therefore considered as having ISM.

The clinical presentation of patients with SM is highly variable. Thus, decisions regarding whether or not to initiate treatment are often complex. Patients with ISM rarely exhibit leukemic transformation and their life expectancy is not significantly different from the age- and sex-matched US population, as demonstrated in a retrospective study by Lim et al. on 342 patients with SM [25]. Thus, the treatment of ISM is mainly symptom-oriented [26]. Lim et al. also identified advanced age (defined as ≥65 years), weight loss, anemia, thrombocytopenia, hypoalbuminemia, and excess bone marrow blasts as independent adverse prognostic factors for survival [25]. Similarly, a retrospective study done by Butterfield and Weiler on 42 elderly patients with SM reported poor survival outcomes in patients with concomitant thrombocytopenia, leukemias, and myelodysplastic syndrome [27]. As our patient was asymptomatic with no poor prognostic factors, he was not treated for ISM on his initial presentation.

With the patient developing esophageal variceal bleed from portal hypertension due to a mast cell infiltrated liver, he was reclassified as having ASM, in which the primary goal of therapy is reducing the mast cell burden. Treatment options that have been studied in nonrandomized trials include hydroxyurea, interferon-alfa with or without prednisone, imatinib, and cladribine. A retrospective study by Lim et al. on 108 patients with SM favored interferon-alfa and cladribine as first line therapy, as hydroxyurea and imatinib were associated with lower overall response rates [28]. Imatinib is relatively ineffective in patients with SM who have a mutation of KIT D816V [29]. This patient therefore was a poor candidate for imatinib, and the lack of randomized controlled trials with evidence favoring one treatment over the others justified the use of hydroxyurea in this patient. A global phase II trial of the use of midostaurin, an oral multikinase inhibitor of both wild-type and D816-mutated KIT, is currently underway with primary results showing high overall response rates with reduction in mast cell burden, a favorable safety profile, as well as improvement in symptoms and quality of life [30]. Seeing this as the largest prospective trial in advanced systemic mastocytosis, midostaurin may become the future standard of care.

In summary, we report the case of a 59-year-old man who initially presented to our clinic with ISM associated with smoldering multiple myeloma. Using the 2008 WHO classification, he was diagnosed with SM-AHNMD. For both diseases, the clinical approach was close follow up until 2 years later when the patient progressed to ASM as evidenced by esophageal variceal bleed secondary to portal hypertension from mast cell infiltration of the liver. The patient was then offered cytoreductive therapy. The association of plasma cell disorders with systemic mastocytosis is rare and the neoplastic cells are believed to be derived from distinct clones. Some postulate that cytokines secreted by the neoplastic mast cells induce the proliferation of plasma cells. The treatment for each disease is planned as if the other did not coexist in the same patient.

## Figures and Tables

**Table 1:** Previously reported cases of SM with an associated plasma cell disorder.

Reference	Age/sex	Diagnosis
Sotlar et al. [4]	70/M	SM and multiple myeloma with secondary amyloidosis
Du et al. [5]	57/F	SM, chronic lymphocytic leukemia, and multiple myeloma
Jain et al. [6]	64/F	ASM and refractory multiple myeloma
Filanovsky et al. [7]	76/M	ISM and smoldering multiple myeloma
Motwani et al. [8]	71/M	SM and multiple myeloma
Stellmacher et al. [9]	51/M	SM and multiple myeloma
Hagen et al. [10]	48/F	SM and multiple myeloma
Pullarkat et al. [11]	84/M	SM and monoclonal gammopathy of undetermined significance

SM: systemic mastocytosis; ISM: indolent systemic mastocytosis; ASM: aggressive systemic mastocytosis.

**Table 2:** Laboratory test results upon initial presentation.

Lab test	Result	Unit
White blood cells	5.3	×10^3^/mm^3^
Hemoglobin	14.5	g/dL
Platelet	202	×10^3^/mm^3^
Blood urea nitrogen	26	mg/dL
Creatinine	1.31	mg/dL
Albumin	3.1	g/dL
Globulin	6.2	g/dL
Calcium	8.8	mg/dL
Serum protein electrophoresis (SPEP) and serum immunofixation	IgG kappa monoclonal protein	
M-spike	3.68	g/dL
